# Parental involvement and association with adolescents’ fruit and vegetable intake at follow-up: Process evaluation results from the multi-component school-based Boost intervention

**DOI:** 10.1186/s12966-016-0435-1

**Published:** 2016-10-26

**Authors:** Sanne Ellegård Jørgensen, Thea Suldrup Jørgensen, Anne Kristine Aarestrup, Pernille Due, Rikke Krølner

**Affiliations:** 1 National Institute of Public Health, University of Southern Denmark, Øster Farimagsgade 5A, 2., 1353 Copenhagen K, Denmark; 2 Council on Health and Disease Prevention, Kristianiagade 12, 2100 Copenhagen Ø, Denmark; 3 Intersectoral Research Unit for Health Services, The Capital Region of Denmark, Bispebjerg Hospital, 23 Bispebjerg Bakke, 2400 Copenhagen NV, Denmark

**Keywords:** Fruit and vegetable intake, Adolescents, School intervention, Parental involvement, Process evaluation

## Abstract

**Background:**

Based on the assumption of parental influence on adolescent behavior, multicomponent school-based dietary interventions often include a parental component. The effect of this intervention component is seldom reported and the evidence is inconsistent. We conducted a systematic process evaluation of the parental component and examined whether the leveal of parental involvement in a large multi-component intervention: the Boost study was associated with adolescents’ fruit and vegetable (FV) intake at follow-up.

**Methods:**

The Boost study was targeting FV intake among 1,175 Danish 7^th^ graders (≈13- year-olds) in the school year 2010/11. The study included a school component: free FV in class and curricular activities; a local community component: fact sheets for sports- and youth clubs; and a parental component: presentation of Boost at a parent-school meeting, 6 newsletters to parents, 3 guided student-parent curricular activities, and a student-parent Boost event. *Study population*: Students whose parent replied to the follow-up survey (*n* = 347). *Data*: Questionnaire data from students, parents and teachers at 20 intervention schools. Process evaluation measures: dose delivered, dose received, appreciation and level of parental involvement. Parental involvement was trichotomized into: low/no (0–2 points), medium (3 points) and high (4–6 points). The association between level of parental involvement and self-reported FV intake (24-h recall), was analyzed using multilevel regression analyses.

**Results:**

The Boost study was presented at a parent-school meeting at all intervention schools. The dose delivered was low to moderate for the three other parental elements. Most parents appreciated the intervention and talked with their child about Boost (83.5 %). High, medium and low parental involvement was found among 30.5 %, 29.6 % and 39.4 % of the students respectively. Parental involvement was highest among women. More men agreed that the parental newsletters provided new information.

Students with a medium and high level of parental involvement ate 47.5 and 95.2 g more FV per day compared to students with low level/no parental involvement (*p* = 0.02).

**Conclusions:**

Students with a high level of parental involvement ate significantly more FV at follow-up compared to students with a low level/no parental involvement. Parental involvement in interventions may improve adolescents’ FV intake if challenges of implementation can be overcome.

**Trial registration:**

ISRCTN11666034. Registered 06/01/2012. Retrospectively registered.

## Background

A high intake of fruit and vegetables (FV) has been associated with a lower risk of lifestyle diseases such as cardiovascular diseases and certain cancers [1–3]. Large proportions of adolescents in Western countries do not meet the World Health Organization’s recommendations of consuming at least 400 g FV daily [4–6] and FV intake tends to decrease during adolescence [7, 8]. Establishing healthy eating habits in childhood is important, as health behaviors established during childhood and adolescence are likely to continue into adulthood [9–14].

Parental knowledge and attitudes, parenting styles, role modeling, and food availability and accessibility have shown to affect children’s dietary behavior [15–17], including FV intake [18–22]. However, the majority of the available literature on determinants of FV intake concerns children in primary schools, while there is less information on how this relates to adolescents. Parents’ food preferences and knowledge affect the availability and accessibility of FV at home [17] and adolescents learn about eating behavior, dieting, cooking etc. by watching others, for instance their parents [20]. This is in line with the finding that FV intake among children and youth is positively associated with parents’ intake of FV [17, 18, 23]. Furthermore, different family related sociodemographic variables have shown to be strong determinants of adolescents’ intake fruit and vegetables [18, 22]. For instance, children and adolescents of parents with low socioeconomic position are more likely to have a poorer diet, characterized by high intake of fats and sugars, and inadequate intake of FV [17, 18, 24, 25]. A review of determinants for fruit and vegetable intake among adolescents shows that girls tend to have a higher or more frequent intake of fruit and/or vegetables than boys [18]. Furthermore, fruit and/or vegetable consumption tend to be lower among children from single parent families than among children living with two adults [18].

As parental influence is regarded essential for adolescent dietary behavior, school-based multicomponent dietary interventions often try to involve parents by including a parental intervention component. Although this is the case, few studies report on the content and results of the parental component [26–29]. Therefore, little is known about how to involve parents successfully in school-based health interventions, resulting in lack of evidence as to the effect of parental involvement on behavioral change [26–29].

The studies which include a parental intervention component have used different approaches to evaluate the implementation and effect of the parental component of the intervention. In a systematic review of 24 studies addressing parental involvement in interventions to improve children’s diet, the authors were unable to conclude upon the effect of parental involvement [28]. Only four studies compared intervention arms with and without a parental component [28]. However, the authors did find patterns suggesting that direct involvement of parents through workshops and cooking classes seems more effective in changing children’s dietary behavior than indirect involvement such as newsletters, homework, and school events [28]. Meanwhile a qualitative study exploring parents’ views on involvement in a school-based obesity intervention indicate that activities such as newsletters and homework are feasible to implement [26]. This finding is supported by a process evaluation of the family component in a school-based nutrition program [30]. Blom-Hoffman et al. [30] succeeded in increasing parental knowledge through an interactive children’s book, however there was no significant effect on children’s FV consumption. The lack of studies addressing the level of parental involvement in relation to the effect of the intervention is also evident in a review by Van Lippevelde and colleages [29]. The review addresses the importance of parental involvement in school-based nutrition and physical activity interventions. The authors find no conclusive evidence of the effect of parental involvement due to the lack of studies addressing the level of parental involvement.

Knowledge regarding the characteristics of parents who are highly engaged in health promotion programs is also sparse [29]. Research on parental participation in students’ schooling shows that family income, education [31, 32], and gender of the child [32] influence parental involvement.

Due to the lack of studies addressing the level and effect of parental involvement, the available evidence on successful parental involvement is scarce and inconclusive. Furthermore, there is a scarcity of literature specific to adolescence with regards to interventions that included effective parent components. Process evaluation of implementation of parental components is critical in order to determine the level of parental involvement in school-based interventions. The process evaluation enables the researcher to distinguish between inadequate implementation of the intervention elements and ineffective intervention elements, thereby qualifying the understanding of any effect of an intervention [33]. The aim of the present study was to:

1. Conduct a systematic process evaluation of the parental component of the Boost intervention by assessing dose delivered, dose received and parental appreciation
2. Explore differences in the characteristics of students with differing levels of parental involvement.
3. Examine whether the level of parental involvement is associated with FV intake among adolescents at follow-up and examine whether this association is modified by family education level, family structure and gender of the adolescent.

## Methods

The Boost study was a national cluster-randomized trial testing the ability of a multi-component school-based intervention to promote FV consumption among Danish 13-year-olds. The multi-component intervention targeted three settings: schools, parents/home and local communities. The intervention was implemented in 7^th^ grade and lasted nine months (September 2010–May 2011). From a list of all 98 Danish municipalities, ten municipalities were randomly selected to join the study, and in each municipality, four schools were randomly selected and invited. After consent to participate the schools in each municipality were randomized into two intervention schools and two control schools [34].

The twenty intervention schools were asked to assign two teachers as local Boost coordinators to facilitate the implementation process of the intervention. The design of the Boost trial is described in detail elsewhere [34].

### The Boost intervention

The Boost intervention involved intervention initiatives at the school level, in the local community and towards parents and the home environment.

The school component involved daily provision of one piece of free fruit or vegetable during school hours, promotion of a pleasant eating environment in class, and curricular activities including a computer tailoring program. The 7^th^ grade teachers were instructed to implement all compulsory activities in the Boost curriculum, but were allowed to adapt them to their local context. The local community component comprised of fact sheets for sports- and youth clubs. The focus of this study is the parental component, which is described in detail below. Further details concerning the development, the theoretical background, the logic model, the design of the full intervention and the ethics of the study are available elsewhere [34].

### The parental component of the Boost intervention

Based on available literature the four elements of the parental component were tailored to influence different determinants of adolescents’ FV intake. The parental component was designed to establish parental support for the Boost study, to increase home availability and accessibility of FV, and to increase parental knowledge and awareness about the importance of FV intake [34]. The parental component comprised four elements:

1. Six *parental newsletters* informing parents about Boost activities at school, and addressing ways to overcome parental barriers for serving FV at home. Approximately every second month during the intervention period, the local Boost coordinators at each school were asked to post a newsletter on the school’s website. We chose to use the school’s website to distribute the newsletters, as this is the primary school-parent communication platform at most Danish schools. The six newsletters addressed each of the following topics:
  Home availability, e.g. variation in types of FV offered
  Home accessibility, e.g. ideas on how to serve more FV
  13-year-olds’ access to FV in their leisure time such as at sports- and youth clubs
  Parental knowledge about FV recommendations, health benefits etc.
  Parental intake of FV
  Perceived parental barriers for making FV available to teenagers at home, e.g. price, time costs, concerns regarding whether FV can satisfy hungry teenagers’ appetite, food preparation skills, family taste preferences, and choosiness of teenagers.
2. Three *guided student*-*parent curricular activities* for the students to conduct at home with their parents. Activity 1: The students were asked to register their own FV intake using a computer tailoring module accessed via the internet. The module generated individually tailored messages to each student and the students were asked to discuss the results with their parents. Activity 2: The students were asked to register the FV eaten by their parents, siblings and by themselves during one week, and to discuss the results with their family. Similarly students were asked to complete a FV taste preference chart with their family and discuss e.g. how they could help each other in the family to eat more FV. Activity 3: Students were asked to register availability of FV in their home and discuss the results with their family. The 7^th^ grade teachers were responsible for assigning the guided student-parent curricular activities to the students.
3. As part of the Boost project week, schools were encouraged to invite parents to a s*tudent*-*parent event* organized by the students. At the student-parent event, students presented results from the Boost curricular activities.
4. In Denmark, a *parent school meeting* is held every year at the beginning of the school year to inform parents about school and curricular activities. The Boost research group attended these meetings to present the Boost intervention and study prior to intervention start. At one school this presentation was conducted by the teachers. In Denmark, these yearly parent school meetings are attended by almost all parents and very often by both mother and father of each child. The meetings are highly valued and are an important information opportunity in the Danish school context. At the meeting, parents were encouraged to take part in the Boost intervention and to support their adolescent in eating FV.

## Data collection

The Boost study collected baseline, first and second follow-up questionnaire surveys among students, parents, teachers, Boost coordinators and school principals in August 2010, May/June 2011 and May/June 2012. Students, teachers, Boost coordinators and school principals completed web-based questionnaires. Students were provided paper questionnaires for both of their parents to complete at home.

### Study sample

In this study, we used data from baseline from students, and from first follow-up from students, parents, teachers and Boost coordinators at the 20 intervention schools. We used data from the intervention schools, only, as we were interested in implementation of the parental component and the importance of different levels of implementation. In the remainder of the paper, first follow-up will be referred to as follow-up.

### Measures

#### Process evaluation measures

The evaluation of the parental component in the Boost study was guided by a systematic process evaluation protocol developed specifically for the Boost study [35]. The protocol outlines the relevant data sources, methods and timing of data collection to ensure that necessary information is recorded about the different parts of the implementation process. In this study, the following process evaluation concepts are used [35, 36].

##### Dose delivered

The amount of the intervention dose that was provided by the Boost research group, by the local Boost coordinators and by the teachers to the parents. At six schools no information was provided from Boost coordinators, and information about number of newsletters uploaded to the school’s website was retrieved from parent data: the highest reported number of newsletters read by any parents was used as a proxy for the number of uploaded newsletters. At the 14 schools with data from Boost coordinators the consistency between the number of uploaded newsletter reported by the Boost coordinators and number of newsletters read by parents was high: At nine schools, the maximum number of newsletters reported by parents was equivalent to the number reported by the Boost coordinators, at four schools the maximum number of newsletters reported by parents was lower than reported by the Boost coordinators and at one school, the maximum number of newsletters reported by parents exceeded the number reported by the Boost coordinator. This could be explained by the fact, that two Boost coordinators were responsible for uploading the newsletters at the school, and only one of them answered the questionnaire at this school.

##### Dose received

The extent to which the parents received and engaged in the intervention components delivered; Dose received was estimated using parent responses e.g. number of newsletters read by parents.

##### Appreciation

Appreciation was based on parental responses to the question “*What did you think of the Boost study all in all*?” and several questions about the usefulness of the parental newsletters.

Table 1 summarizes process evaluation measures including data source, categorization, and time of assessment.

**Table 1:** Description of process evaluation measures, outcomes and covariates included in the analyses

Intervention component or item	Respondent	Question/measure	Response categories/codes	Range (continuous variables) and categories (categorical) of variables used in the results and analyses	Time of assessment
Process evaluations measure: dose delivered					
Parental newsletter	School coordinators	“During this school year, Boost emailed six parental newsletters for the Boost coordinator to post them on the school’s website. How many of these were posted?”	1) None of the newsletters 2) One newsletter 3) Two newsletters 4) Three newsletters 5) Four newsletters 6) Five newsletters 7) Six newsletters	1) 0/missing 2) 1–3 newsletters 3) 4–6 newsletters	Follow-up
Student/parent curricular activities	Students	Students were presented with a short description of each Boost curricular activity and were asked to rate how much the liked each of the activities.	I did not do this activity or Rating the activity from 0 (worst) to 10 (best)	Each activity rated by the student counted as one activity delivered to the student. Guided student parent activities: 0–3	Follow-up
Students/parent Boost event	Students	Students were presented with a short description of each Boost curricular activity and were asked to rate how much the liked each of the activities.	I did not do this activity or Rating the activity from 0 (worst) to 10 (best)	Student-parent event: yes/no	Follow-up
The parent-school meeting	Boost research team	The Boost research team presented the intervention at 19 og the 20 intervention schools. At the last school, the teacher reported that boost was presented at the meeting.		Presentation of the Boost study at the parent-school meeting: yes/no	Follow-up
Process evaluation measure: dose received					
Parental newsletter	Parents	“Have you seen that there have been parental newsletters from the Boost study on the school’s website during 7^th^ grade?” “How many of them (the newsletters) have you read”	Yes No I do not have access to the school’s website 1) None of the newsletters 2) One newsletter 3) Two newsletters 4) Three newsletters 5) Four newsletters 6) Five newsletters 7) Six newsletters	1) Yes 2) No/I do not have access to the school’s website 1) 0/missing 2) 1–3 newsletters 3) 4–6 newsletters	Follow-up
Student/parent curricular activities	Parents	Parents were presented with a short description of each of the guided student-parent curricular activity and were asked if they completed or discussed this activity together with their child. E.g. “The activity “availability in the family” is a Boost homework activity, where the students should register availability of FV in their home. Have you, together with your child, completed or discussed this activity?”	Example of response categories for the activity concerning availability of FV in the family. Similar response categories were given for the other guided student-parent curricular activity. 1) I do not know of this activity 2) No, we did not do the activity together 3) Yes, we talked about the availability of FV at home 4) Yes, we discussed the questions in the Boost student workbook.	Each activity with at least one parent replying 3 or 4 counted as one activity received. The sum of activities received by parents ranged from 0 to 3 activities.	Follow-up
Students/parent Boost event	Parents	“Have you participated in a student-parent Boost event, where your child’s school class presented some of the activities they have done in the Boost project?”	1) I do not know if there has been such an event 2) The school did not host such an event 3) No, I did not participate 4) No, but another member of the family participated 5) Yes, I participated	At least one parent participated: 1) Yes: One or both parents responded “Yes, I participated” 2) No: None of the parents responded “Yes, I participated”	Follow-up
The parent-school meeting	Parents	“Did you learn about the Boost study at the first parent-school meeting during 7^th^ grade?”	1) I do not know of any parent-school meeting/there was no parent-school meeting 2) No, I did not participate in the meeting 3) No, there was no information about the Boost study at the meeting 4) Yes, a teacher/Boost team member introduced the study 5) Do not know	At least one parent heard about boost at the meeting: 1) Yes: One or both parents responded “Yes, a teacher/Boost team member introduced the study”) 2) No: None of the parents responded “Yes, a teacher/Boost team member introduced the study”	Follow-up
Process evaluation measure: appreciation					
Overall	Parents	“What did you think of the Boost study all in all?”	1) I liked it very much 2) I liked it somewhat 3) I did not like it 4) I do not know	1) I liked it very much 2) I liked it somewhat 3) I did not like it 4) I do not know	Follow-up
Parental newsletters	Parents	Parents were asked to rate their level of agreement with four statements about the parental newsletters. “The newsletters have given me tips on how I can serve more fruits to my family “ “The newsletters have given me tips on how I can serve more vegetables to my family” “The newsletters informed me of things that I did not already know” “The newsletters reminded me of things I already knew”	1) Fully agree 2) Agree 3) Neither agree nor disagree 4) Disagree 5) Totally disagree	1) Fully agree/agree 2) Neither agree nor disagree/disagree/totally disagree	Follow-up
Outcome measure					
Student-reported total daily intake of FV	Students	24-h recall questionnaire based on detailed questions on yesterday’s intake of FV at three different times of the day. The fruit measure included max 100 g juice. Potatoes were excluded. Exclusion of outliers >1200 g/d	Number of portions and pieces of different fruits and vegetables	0-1200 g	Follow-up
Covariate: FV intake at baseline					
Student-reported total daily intake of FV	Students	24-h recall questionnaire based on detailed questions on yesterday’s intake of FV at three different times of the day. The fruit measure included max 100 g juice. Potatoes were excluded. Exclusion of outliers >1200 g/d	Number of portions and pieces of different fruits and vegetables	0-1200 g	Baseline
Covariate: Dose delivered of other intervention components					
Provision of FV at school	Teachers	“How often do you cut up FV when students eat FV during your lessons?”	1) Every time 2) Most times 3) Some times 4) Seldom 5) Never	School-level dose: proportion of teachers at each school cutting up FV every time/most times students eat FV in class 1) ≤50 % (reference group) 2) >50 %	Follow-up
Curricular component	Teachers	“Which of the Boost curricular activities from the teacher manual mentioned below did you teach during the Boost intervention period September 2010–May 2011?”	List of all Boost curricular activities to tick off (listed by number and name consistent with teacher manuals)	School-level dose: average number of Boost curricular activities delivered by teachers at each school Low (0–3.8) (reference group) Medium (3.9–6.7) High (≥6.8)	Follow-up
Covariate: sociodemographic					
Gender	Students	“Are you a boy or a girl?”	1) Boy 2) Girl	1) Boy 2) Girl	Baseline
Family educational level	Parents	“Which school education do you have?” “Which vocational education do you have?” (If you have more than one, please tick off the highest level of education)	Based on completed education, mothers and fathers were categorized into one of seven educational categories using national coding principles. 1) Enrolled in education 2) Primary school 3) Manual education 4) Low theoretical education 5) Medium high theoretical 6) Education 7) High theoretical education	Family educational level was based on the highest ranking parent. Unclassifiable parents were excluded. 1) High education: 7 2) Medium high education: 6 3) Low education/none: 1–4 (reference group)	Baseline
Family structure	Students	“Who do you live with? If you live in more than one place or with more adults, you may give several replies”	1) My mom, who lives without a partner 2) My dad, who lives without a partner 3) My mom and her new partner 4) My dad and his new partner 5) Both my mom and dad all the time 6) My grandmother 7) My grandfather 8) In an orphanage or foster home 9) With other adults (write whom)	In the case of multiple responses, living with both mother and father overruled other responses, and living with mother overruled living with father. 1) Live with two adults:3,4,5 2) Live in single parent family:1,2 Only three students have responded 6–9 and these were coded excluded from the analyses.	Baseline
Mothers country of birth	Students	“Is your mother born in Denmark?” “In what country is your mother born?”	1) Yes 2) No 3) Don’t know Drop down list of all countries.	1) Denmark 2) Other country	Baseline

#### Measure of parental involvement

The measure of parental involvement was based on the measures of dose received of all four parental elements (see Table 1) and the parental responses to the question ‘*Did you speak with your child about the Boost study*?’ This last question was included as an indicator of parental interest in the Boost study. The four elements of the parental component were tailored to different determinants of adolescents’ FV intake e.g. home availability of FV and social determinants such as parent modeling, parental intake of FV, parental knowledge etc. In order to be categorized as having a high level of parental involvement it was required that at least one of the parents had participated in three out of four elements of the intervention. If two parent surveys were received, the dose received was based on the highest dose reported in the two surveys. Participation of at least one parent was considered sufficient as a representative of the student’s home environment. Participation was assigned points as follows: Read 1–3 *newsletters* = 1 point, read 4–6 *newsletters* = 2 points, participated in and heard about the Boost study at a *parent*-*school meeting* = 1 point, participated in 1–3 *guided student*-*parent activities* = 1 point, participated in the *student*-*parent event* = 1 point, *spoken with child about the Boost study* often or sometimes = 1 point. The parental newsletters were the most extensive of the four elements. Each newsletter addressed a new topic and the newsletters were delivered throughout the intervention period. Thus, reading 4–6 newsletters yields 2 points, as parents who had read at least four newsletters were exposed to at least 4 of the 6 topics throughout at least half the intervention period. The index of parental involvement ranged from 0–6 points and was trichotomzed into: low/no (0–2 points), medium (3 points) and high (4–6 points). This was done in order to investigate a possible dose-response relationship between dose received by parents and students FV intake at follow-up. The number of categories was limited by the number of parent responses. Including more categories would have compromised the statistical power, especially in the subgroup analyses.

We explored the level of parental involvement both at the student level and at the parent level. At the parental level we used all parental responses, if available two parents per students. This variable was used to look at the characteristics of participating parents. Level of parental involvement at the student level was based on parental participation of least one parent per student. The variable for parental involvement used in the analyses of associations is at the student level, as the outcome of interest is at the student level.

#### Measure of F&V intake

Students’ daily FV intake was measured at baseline and follow-up in grams/day by use of the validated pre-coded 24-h recall questionnaire from the Pro Children study [37]. Students reported how many pieces/portions of specific FV they consumed before school, at school, and after school on the previous day. Based on standardized guidelines, pieces and portions of FV were converted into grams. One piece or portion of FV corresponds to approximately 100 g [37, 38]. In agreement with Danish dietary recommendations, the fruit measure included a maximum 100 g of 100 % natural juice regardless of the number of glasses consumed [39]. The vegetable measure did not include potatoes.

#### Measures of covariates

The analyses of the association between level of parental involvement and adolescents’ FV intake at follow-up were adjusted for students’ self-reported total daily FV intake in grams at baseline.

Potential confounding and effect modifying factors were identified a priori based on theoretical and empirical consideration [34]. These include the following variables which are all further described in Table 1.

- Dose delivered of other components of the Boost intervention: a) Provision of FV b) Dose delivered of the curricular component
- Gender.
- Family educational level, determined by the highest completed education of the highest ranking parent, and was coded into three educational levels: high, medium and low/no.
- Family structure: students were asked ‘Who do you live with’: a) mother, b) father, c) mother and her new partner, d) father and his new partner, e) mother and father, f) grandmother, g) grandfather, h) foster family or i) other adult. In the case of multiple responses, living with mother and father overruled other responses, and living with mother overruled living with father. The students were categorized into two groups: ‘live with two adults’ or ‘live in a single parent family’,
- Mother’s country of birth was categorized as ‘Denmark’ or ‘other country’.

### Statistical analyses

Chi-square tests were used to detect differences between students with and without parent responses at follow-up. We used descriptive statistics to assess dose delivered, dose received and appreciation of the parental component. To account for the nested design, the association between level of parental involvement and students’ FV intake at the end of intervention was examined using multi-level general linear modeling including school-, class-, and student level. The analyses were adjusted for students’ FV intake at baseline, family educational level, family structure and mother’s country of birth. To account for the effect of the other elements of the Boost intervention the analyses were also adjusted for dose delivered of the other component of the Boost intervention. Furthermore, potential effect modifying factors were examined by 1) including interaction terms between level of parental involvement and gender, level of parental involvement and family education level, and level of parental involvement and family structure in three separate analyses and 2) analyses stratified by the potential effect modifiers. To examine the implications of changing cut-points for outliers of FV intake at follow-up, we performed sensitivity analyses using the cut-points >1,000 g and >1,400 g of FV daily.

Model assumptions were considered to be satisfied based on visual inspection of residual plots and QQ-plots. We tested for linearity between parental involvement level and FV intake at follow-up by visual inspection of scatter plots. We identified weak collinearity between level of parental involvement and dose delivered of the school component, curricular activities and a pleasant eating environment (Spearman’s correlation coefficients <0.2). We used the statistical software package SAS version 9.3 for all analyses. A 0.05 significance level was chosen a priori.

### Ethics

The Boost study adheres to all Danish ethical standards and the Declaration of Helsinki and is approved by the Danish Data Protection Agency (J.nr. 2010-54-0974). School boards (parental representatives), school principals, teachers and students board all have approved of the study and everyone involved were informed of their ability to withdraw from the study at any point in time. Parents could have their child’s questionnaire excluded from the database by ticking a box on the front page of the parent questionnaire. At the 20 intervention schools, 11 parents at baseline and 12 parents at follow-up wanted their child’s questionnaire excluded. Responses were treated anonymously and confidentially. For more information on the ethic approval of the Boost study see: http://www.isrctn.com/ISRCTN11666034.

## Results

### Response rates and study population

The Boost study involved 1,175 students at intervention schools, of whom 1,121 completed the questionnaire at baseline (response rate: 95.4 %) and 1,060 completed the follow-up questionnaire (response rate: 90.7 %). At the 20 intervention schools, 658 parents of 423 students completed the follow-up questionnaire, corresponding to 39.9 % of the students having at least one parent response. Among all 7^th^ grade teachers, 114 completed the follow-up questionnaire (response rate: 45 %). All 20 intervention schools were represented by at least one teacher response. Questionnaires were completed by Boost coordinators at 14 of the 20 intervention schools.

The analyses of the association between level of parental involvement and students’ FV intake included data from students who had completed baseline and follow-up surveys (*N* = 991) and had a parent reply at follow-up (*N* = 423). Furthermore, the study population was restricted to students who had completed a 24-h dietary recall questionnaire, used to estimate the total daily FV intake (*N* = 404). We defined a cut-point of 1,200 g as the highest plausible daily FV intake based on recommendations from previous studies [40]. This resulted in exclusion of 36 and 21 students at baseline and follow-up, respectively. Missing data were excluded from the analyses. The final study sample in analyses of the association between the level of parental involvement and FV intake among adolescents at follow-up comprised 347 students.

Table 2 shows the characteristics of the study population. For students at intervention schools, the mean FV intake was 405.6 g at baseline (median: 350.0, SD: 289.1)/day and 422.4 g at follow-up (median: 380.0, SD: 290.0)/day.

**Table 2:** Sociodemographic characteristic of the study population and distribution of daily FV intake at baseline and follow-up

Individual-level characteristics	*n* (%)	FV intake at baseline, grams Mean (SD)	FV intake at follow-up, grams Mean (SD)	Missing (*n*)
All students	347	405.6 (289.1)	422.4, (290.0)	0
Gender				
Boys	178 (51.3)	371.1 (292.9)	378.3 (266.9)	0
Girls	169 (48.7)	442.0 (281.4)	468.8 (306.5)	
Family educational level				
High	67 (19.3)	466.9 (295.4)	465.9 (328.2)	2
Medium	136 (39.2)	435.5 (291.4)	438.8 (272.9)	
Low/no	142 (40.9)	353.4 (274.9)	389.5 (285.7)	
Family structure				
Live with two adults	306 (88.2)	409.4 (293.4)	429.0, (295.3)	0
Live in single parent family	41 (11.8)	377.5 (256.3)	372.5 (244.9)	
Mother’s country of birth				
Denmark	329 (94.8)	408.0 (285.3)	426.1 (292.2)	3
Other country	15 (4.3)	397.9 (376.8)	345.3 (189.9)	

### Attrition analysis

Students with at least one parent reply at follow-up were more likely to have a parent reply at baseline (85.1 % versus 41.7 %, *P* < 0.0001), have parents born in Denmark (e.g. mother born in Denmark, 95.2 % versus 79.7 %, *P* < 0.0001) and live in a family with two adults (87.1 % versus 80.9 %, *P* = 0.009). There was no significant difference between students with and without a parent reply at follow-up with regards to family educational level, gender of the child or number of siblings. Furthermore, students with at least one parent reply at follow-up had a higher FV intake at follow-up (mean/median 429.2g/380 g versus 392.4g/330 g, *P* = 0.05) compared to students with no parent reply at follow-up. The same tendency was seen for mean and median FV-intake at baseline; however, the difference for this measure was not significant (mean/median: 414.0/370 g versus 381.1/314 g, *P* = 0.09).

### Process evaluation of the parental component

#### Dose delivered

Dose delivered is reported as the percentage of students to whom the intervention elements were delivered.

Four to six *newsletters* were uploaded at the school’s website at 14 schools, corresponding to three-quarters of the 423 students with a parent reply. At five schools one to three newsletters were uploaded and at one school, none of the newsletters were uploaded according to Boost coordinators. The most frequently mentioned reasons for not uploading newsletters were technical problems and forgetfulness of Boost coordinators. Two-thirds of the students reported that they completed between one and three of the *guided students*-*parent curricular activities* (Table 3). The lowest dose delivered was found for the *student*-*parent Boost event*, which only 32.6 % of the students attended. The Boost study was introduced at a *parent*-*school meeting* at all intervention schools (Table 3).

**Table 3:** Distribution of students according to dose delivered and dose received of the parental component (*n* = 423)

Intervention item	Dose delivered (reported by students, Boost-coordinators and the Boost research group) % (*n*)	Dose received (reported by parents) % (n)
Seen the newsletters at the school’s website		78.5 (332)
Newsletters uploaded by Boost coordinators/read by parents		
0/missing	1.9 [8]	35.5 (150)
1–3	23.2 (98)	53.0 (224)
4–6	74.9 (317)	11.6 [49]
Guided student-parent curricular activities		
0/missing	33.3 (141)	70.7 (299)
1	19.4 (82)	15.4 (65)
2	19.4 (82)	8.5 [35]
3	27.9 (118)	5.4 [23]
Student-parent Boost event	32.6 (138)	15.4 (65)
Parent-school meeting	100 (423)	76.8 (325)
Talked with child about the Boost study often og sometimes	–	83.5 (353)

#### Dose received

Dose received of the parental component is reported as the percentage of students whose parents engaged in the intervention components (at least one parent per student).

More than 78 % students had at least one parent, who had seen the *newsletters* at the school’s website, and 64.6 % of the students had one or more parents who had read at least one *newsletter*. Among 29.3 % of the students, at least one parent had participated in the *guided students*-*parent curricular activities*. Parents of 15.4 % of the students had participated in a *student*-*parent Boost event*. For more than three-quarters of the students, at least one parent had attended the *parent*-*school meeting* (Table 3). For 83.5 % of the students at least one parent reported talking to them about the Boost study often or sometimes.

#### Appreciation

Of the 658 parents who responded to the follow-up questionnaire, 58.4 % liked the Boost study very much, 24.2 % liked it somewhat, 1.4 % did not like the Boost study at all and 14.4 % did not know what to think about Boost. More women (64.6 %) than men (48.2 %) liked the Boost study very much and more men (19.1 %) than women (11.4 %) answered ‘do not know’ to the question about their overall impression of the Boost study (*p* = 0.001). There were no differences in the overall impression of the Boost study with regards to parents’ educational level or country of birth.

Table 4 shows parents’ appreciation of the parental newsletter. Twice as many fathers/stepfathers as mothers/stepmothers, and more parents with low educational level compared to high education level agreed with the statement ‘*the parental newsletters provided new information*’. Furthermore, fewer parents with high education found that the newsletters provided useful serving tips compared to parents with medium or low educational level (not significant (NS)).

**Table 4:** Appreciation: Proportion and characteristics of parents who agree/fully agree with four questions about the parental newsletters

		Agree/fully agree	Chi square
	*n*	%	*p*
The newsletters provided new information			
All parents	314	19.4	
Gender			
Mother/stepmother	229	15.3	<0.01*
Father/stepfather	84	29.8	
Education level			
High	43	14.0	0.03*
Medium	118	14.4	
Low/none	144	25.0	
The newsletters reminded me of things I already knew			
All parents	315	60.6	
Gender			
Mother/stepmother	231	61.5	0.11
Father/stepfather	83	57.8	
Education level			
High	43	58.1	0.55
Medium	120	65.0	
Low/none	144	60.4	
The newsletters provided useful serving tips for fruits			
All parents	315	29.8	
Gender			
Mother/stepmother	230	30.0	0.30
Father/stepfather	84	29.8	
Education level			
High	43	23.3	0.14
Medium	119	30.3	
Low/none	144	30.9	
The newsletters provided useful serving tips for vegetables			
All parents	314	28.7	
Gender			
Mother/stepmother	229	28.8	0.06
Father/stepfather	84	28.6	
Education level			
High	43	23.3	0.34
Medium	118	27.1	
Low/none	144	29.9	

*Significant difference

### Characteristics of students with participating parents

There were no significant socio-demographic differences between students with low/no, medium and high level of parental involvement (Table 5). However, there was a tendency towards a lower level of parental involvement among students from single parent families compared to students from families with two adults (NS). Furthermore, 46.4 % of the students from families with low educational level had low/no level of parental involvement compared to 31.7 % of students from families with high educational level (NS).

**Table 5:** Socio-demographic characteristics of students according to level of parental involvement

	Level of parental involvement				
Characteristics	*N*	High %	Medium %	Low/no %	Chi square *p*
Gender					
Girl	214	31.8	27.6	40.6	0.65
Boy	209	29.2	31.6	39.2	
Family educational level					
High	82	30.5	37.8	31.7	0.13
Medium	159	33.3	29.6	37.1	
Low/none	179	27.4	26.3	46.4	
Family structure					
Live with two adults	363	31.4	30.0	38.6	0.18
Live in single parent family	54	20.4	29.6	50.0	
Mother’s country of birth					
Denmark	394	30.5	29.7	39.9	*
Other country	20	25.0	30.0	45.0	

*Sample size too small to test

At the parental level, using all 658 parents responses, a high involvement was found among more women than men (25.3 % versus 9.6 %; *p* < *0.001*). There was no significant educational difference between parents with high, medium and low/no level of involvement (data not shown).

#### Level of parental involvement

Level of parental involvement was based on dose received reported by parents and whether parents had spoken with their child about the Boost study. At the student level, parental involvement was high, medium and low for 30.5 %, 29.6 % and 39.4 % of the 423 students respectively. At the parental level, based on all 658 parents who responded at the follow-up survey, high, medium and low level of involvement was found for 19.3 %, 26.3 % and 54.4 % of the parents. A larger proportion of parents with high involvement level liked the Boost study very much (78.0 %), compared to parents with medium (67.6 %) and low (46.9 %) involvement (*p* = <.0001).

### Association between level of parental involvement and adolescents’ fruit and vegetable intake

Level of parental involvement in the Boost intervention was significantly associated with adolescents’ FV intake at follow-up. Among the 347 students included in the analysis, students with a medium and high level of parental involvement ate 47.5 (SE: 33.7) and 95.2 (SE: 34.3) grams more FV daily compared to students with low/no level of parental involvement (*p* = *0.02*) (Table 6) when we adjusted for the other intervention components. These results indicate a dose-response association, where adolescents’ FV intake increases with increasing parental involvement.

**Table 6:** Association between level of parental involvement and students’ FV intake, multilevel linear regression analyses, *n* = 347

Stratifying variable	Level of parental involvement	Adjusted^a^ multilevel analysis		
		Estimate (grams/day)	SE	*p*-value
	Low/no (reference)	0		0.02*
	Medium	47.5	33.7	
	High	95.2	34.3	
Gender				
Girls	Low/no (reference)	0		0.03*
	Medium	14.6	55.2	
	High	130.9	53.1	
Boys	Low/no (reference)	0		0.03*
	Medium	101.3	43.2	
	High	97.2	44.9	
Family educational level				
High	Low/no (reference)	0		0.97
	Medium	20.9	94.8	
	High	5.0	94.8	
Medium	Low/no (reference)	0		<0.01*
	Medium	126.1	51.7	
	High	191.6	58.8	
Low	Low/no (reference)	0		0.37
	Medium	−29.6	51.7	
	High	50.8	51.8	
Family structure				
Live with two adults	Low/no (reference)	0		0.13
	Medium	33.5	37.3	
	High	75.7	37.4	
Live in single parent family	Low/no (reference)	0		<0.01*
	Medium	208.0	66.9	
	High	390.8	73.0	

^a^Adjusted for baseline FV intake, family education level, family structure, mother’s country of birth and dose delivered of other intervention components

^*^Significant associations (*p* ≤ 0.05)

None of the effect modifiers were significant when introduced into the model as interaction terms with level of parental involvement. Results from the stratified analyses indicated that among girls, the mean difference in FV intake was larger between high and low level of parental involvement, than between medium and low level of parental involvement. Boys with both medium and high level of parental involvement ate approximately 100 g more FV per day compared to boys with low/no level of parental involvement. In analyses stratified by family educational level, the association was only significant for students of medium family educational level. The greatest difference between high and low/no level of parental involvement was seen among students from single parent families (390.8 g more, SE: 73.0).

## Discussion

This study demonstrates that involving parents in multi-component school interventions may improve the FV intake among adolescents. The level of parental involvement in the Boost study was positively associated with students’ FV intake at the end of the intervention, when controlled for implementation of other components in the intervention. Sensitivity analyses revealed that changing the cut point for FV consumption did not change the results markedly.

The process evaluation showed variation in dose delivered of the four elements of the parental component. There was a high dose delivered of the introduction to the Boost study at the parent-school meetings, a moderate dose delivered of the parental newsletters and a low dose delivered of the guided student-parent curricular activities and the student-parent event. The majority of the parents answering the follow-up questionnaire appreciated the intervention. In relation to the characteristics of participating parents, parental involvement was greatest among mothers/stepmothers compared to fathers/stepfathers; but more fathers agreed that the parental newsletters provided new information. Also, more parents with low educational level agreed that the parental newsletters provided new information.

Several reviews have attempted to investigate the effectiveness of parental involvement in health promoting school intervention [28, 29]. Nevertheless, due to the lack of studies that separately evaluate the parent component, evidence for the added effect of parental involvement remain inconclusive.

In line with the findings of the present study, the “Pro Children study” found the greatest increase in vegetable intake among children with high parental involvement, when comparing with children with medium and low parental involvement [27]. The “Pro Children study” found no effect of parental involvement on fruit consumption [27]. A FV intervention study among children aged eight to nine years found that children with high or medium level parental involvement consumed more FV than children of parents with low involvement (NS) [41].

Free provision of one piece of FV at class on a daily basis was an important component of the Boost intervention. It is therefore plausible that part of the increase in FV intake observed in this study can be explained by an increased FV consumption during school hours. As we only included data from interventions schools in this process evaluation study, all students were exposed to free FV at class on a daily basis. Furthermore we included FV provision as a confounder in the analyses by adjusting for how often the teachers cut up the delivered FV. The association between level of parental involvement and adolescents’ FV intake at follow-up indicates a greater increase in intake among those students at intervention schools who experienced high parental involvement compared to students with low parental involvement.

The association between level of parental involvement and adolescents’ FV intake was not modified by family education level, family structure or gender of the adolescent. The insignificant interaction terms indicate that the association is preserved across gender and socioeconomic subgroups. The stratified analyses should therefore be considered explorative and interpreted with caution. The stratified analyses might indicate, that effect among girls requires a higher level of parental involvement than among boys. Furthermore, the stratified analyses could indicate a dose-response association between level of parental involvement and FV intake among students with medium family educational level, but not in students with low and high family educational level. It could be argued that the lack of association among students with high family educational level might result from a ceiling effect concerning students’ FV intake. However, the intake in this age group still needs to improve to reach the national and international recommendations. It has been suggested that health promoting interventions aimed at individual factors might widen social inequality as these interventions require more individual resources and effort to succeed compared to interventions targeting the environment [42, 43]. This may explain the lack of association among students with low family educational level. Parents from low socio economic position may be less receptive to the parental component in the Boost intervention and may not possess the resources necessary to act upon the knowledge acquired from the Boost intervention. Other studies exploring socioeconomically differential effects of health behavior interventions show inconsistent results [44, 45].

The few existing process evaluation studies of parental involvement in school-based FV interventions also show low to moderate levels of parental involvement [27, 41, 46, 47]. Lack of time has been identified as the most common barrier for parental involvement in school-based interventions [26, 48]. As described in the introduction, direct parental involvement like cooking classes has been argued to be more effective ways of influencing and improving adolescents FV intake [28]. However, the Boost intervention was involving parents by use of indirect methods such as newsletters etc. This was done mainly to increase the likelihood of sustainability of the intervention. In a study by Kipping et al. [26] parents were less likely to participate in activities situated at the school due to lack of time. In the present study, two intervention activities were situated at the school: The parent-school meeting, which had high levels of dose delivered and received, and the student-parent Boost event, which had very poor levels of dose delivered and dose received. The parent-school meeting is a well-known, preexisting informational activity at all schools. It takes place every year at the beginning of the school year in every school class at all grades. This means that the parents were used to take part in these meetings, whereas the student-parent Boost event was an extra intervention activity requiring extra time from both teachers and parents. In some school classes this event was not held. Both of these factors may contribute to the explanation of the difference in parents’ participation in the two events. A qualitative process evaluation of the parental involvement in a school-based obesity intervention also showed promising results for parental involvement using existing school setting elements [26]. Kipping et al. [26] found that information about the intervention incorporated into existing school newsletters was well implemented by teachers and positively received by parents. Other studies indicate that home activities encouraging interaction between parent and child is appreciated by parents and seem achievable to implement [26, 30, 47, 48]. This is in contrast with the findings of our study. Only one-third of the students participated in the guided student-parent curricular activities and only 5.4 % of the students had parents who participated in all three activities. This discrepancy might be due to the fact, that the Boost study involved teenagers (13-year-olds) whereas the other studies targeted younger children (kindergarten to 5th grade). Parental involvement in homework is more likely to be an existing routine among younger age groups compared to the older participants in the Boost intervention. The teachers were asked to fill in a logbook of the Boost activities. However, very few teachers did this and therefore we do not have the necessary information available to go deeper into their experiences with the specific curricular activities and thereby clarify whether the use of guided student-parent activities were not suitable for this age group. The Boost intervention targeted teenagers, who are a challenging target group for dietary interventions. During the teen years adolescents are going through a transition period where they start to seek independence and detachment from parents and spend more time with friends. Teenagers have increased opportunities for eating unhealthily as they are often allowed to leave school during breaks; they start spending more time and meals together with their friends, and have their own money to spend, all limiting their parents’ influence as role models, providers, and gatekeepers of their dietary behavior [19, 49]. Another contributing issue to the low level of implementation of the guided student-parent curricular activities may be the high level of working mothers in Denmark (98 %) which makes it difficult to compare internationally on home related issues requiring parental time, when Danish parents simply spend less time at home with their children.

The parental component of the Boost intervention targeted both mothers and fathers. In the planning of the intervention, fathers and parents of low socioeconomic position were identified as target groups most in need of intervention due to their lower FV consumption levels and their influence as role models for the adolescents. Of the parents engaged in the intervention, more mothers than fathers appreciated the Boost study. Moreover, high level of parental involvement seemed to be more frequent among students with high family educational level and among students living with two adults (NS). Wind et al. [27] also showed higher parental involvement among students living with two adults. Although parental involvement in the Boost intervention was low among fathers, the newsletters seemed to be useful to them as larger proportions of fathers agreed that the newsletters provided new information, compared to mothers.

### Study limitations and strengths

The attrition analyses revealed that a selective group of parents completed the follow-up questionnaire and that the study sample may not be fully representative. For instance, the reported dose received of the parental component may be overestimated in the study sample if the parents who completed the follow-up questionnaire were more likely to have engaged in the intervention compared to non-responding parents. However, the potential selection bias is not likely to affect the association between level of parental involvement and FV intake.

Another limitation is that parents and children might be inclined to give socially desirable answers, which may lead to misclassification. In this study, potential misclassification of e.g. level of parental involvement due to social desirability bias is thought to be non-differential, as it is not dependent on the FV intake reported by students. As a result, the association might be biased towards the null. To minimize social desirability bias among students, the Boost research group emphasized that the questionnaire was not a test and that there were no right or wrong answers. Students were encouraged to answer as honest as possible. In the development of the Boost study, the scarcity of studies on effective involvement of parents in interventions specifically targeting the age group of adolescents has challenged the design of the parental component. In the analyses, multiple testing should be considered a limitation. Furthermore, the index of parental involvement was developed specifically for this study, as well as the measures used to assess dose delivered, dose received and appreciation and thus the validity etc. is unknown.

Important strengths of this study include high response rates among students, information on parental involvement from both mothers and fathers and the use of a validated 24-h recall questionnaire for assessment of FV intake. The 24-h recall gives a valid assessment of group-level mean intake [37]. The Boost study was based on a systematic theory- and evidence-based planning procedure [34] and the process evaluation was guided by a thorough and comprehensive process evaluation protocol [35]. Moreover, this is one of the few studies that provide a thorough systematic process evaluation of parental involvement in a school-based FV intervention, to assess the level of parental involvement and to evaluate the association with adolescents’ FV intake.

## Conclusions

The process evaluation of the parental component in the Boost study indicates that: 1) parents appreciated the Boost study overall, 2) using existing structures in the school such as parent-school meetings may facilitate successful involvement of parents in interventions, and 3) low delivery of parental intervention components is a major barrier for parental involvement in school-based interventions. Thus, the process evaluation confirms that involving parents in school-based health interventions is difficult. We found no socio-demographic differences between students with low/no, medium and high level of parental involvement. Nevertheless, we identified a dose-response association with increasing FV intake by higher level of parental involvement. This is promising, as even moderate involvement of parents in interventions seems to influence the FV intake of adolescents.

Future studies should explore the potential of incorporating parental components into existing structures and systems in the school to overcome barriers for parental involvement and teachers’ implementation. The results of this process evaluation also call for studies examining specific barriers for involvement among different subgroups of parents, e.g. fathers and for more insight into specific strategies suitable for the involvement of parents in interventions targeting this age group. Moreover, in order to identify important mediators of the association, future studies should examine the association between parental involvement and the specific determinants of adolescents’ FV intake, which the parental component have been tailored to affect.

## Abbreviations

FV: Fruit and vegetables
NS: Not significant
